# Sensitivities and Dependencies of *BRAF* Mutant Colorectal Cancer Cell Lines with or without *PIK3CA* Mutations for Discovery of Vulnerabilities with Therapeutic Potential

**DOI:** 10.3390/medicina58101498

**Published:** 2022-10-21

**Authors:** Ioannis A. Voutsadakis

**Affiliations:** 1 Algoma District Cancer Program, Sault Area Hospital, 750 Great Northern Road, Sault Ste. Marie, ON P6B 0A8, Canada; ivoutsadakis@yahoo.com or ivoutsadakis@nosm.ca; 2 Section of Internal Medicine, Division of Clinical Sciences, Northern Ontario School of Medicine, Sudbury, ON P3E 2C6, Canada

**Keywords:** colon cancer, cell line models, dependencies, targeted therapy, signal transduction

## Abstract

*Background:* Colorectal cancer represents a common malignancy and remains incurable in the metastatic stage. Identification of molecular alterations that are present in colorectal cancer has led to the introduction of targeted therapies that improve outcomes. *BRAF* and *PIK3CA* mutations are observed in a subset of colorectal cancers. Colorectal cancers bearing *BRAF* mutations may be treated with specific BRAF inhibitors. These drugs benefit patients with *BRAF* mutant colorectal cancers but responses are rather brief, and progression is the rule. In contrast, no PI3K inhibitors have proven successful yet in the disease. Thus, new treatments to supplement the currently available drugs would be welcome to further improve survival. *Methods:* Profiled colorectal cancer cell lines from the Cancer Cell Line Encyclopedia (CCLE) were examined for *BRAF* and *PIK3CA* mutations and were interrogated for molecular characteristics and concomitant alterations that mirror clinical sample alterations. The Genomics of Drug Sensitivity in Cancer (GDSC) project was used for determination of drug sensitivities of *BRAF* mutated colorectal cell lines with or without concomitant *PIK3CA* mutations. The Cancer Dependency Map project served as the basis for identification of molecular dependencies and vulnerabilities in these cell lines. *Results:* CCLE includes 84 colorectal cancer cell lines, which recapitulate the molecular landscape of colorectal cancer. Of these, 23 and 24 cell lines possess *BRAF* and *PIK3CA* mutations, respectively. Seven *BRAF* mutant cell lines have V600E mutations and 14 *PIK3CA* mutant cell lines have hotspot helical or kinase domain mutations. V600E *BRAF* mutant cell lines with or without hotspot *PIK3CA* mutations are heterogeneous in their MSI status and mimic colorectal cancer tissues in other prevalent abnormalities including *APC* and *TP53* mutations. Essential genes for survival include *CTNNB1*, *WRN*, and pyrimidine metabolism enzyme *CAD*. Besides *BRAF* mutations, BRAF inhibitor sensitivity in colorectal cancer cell lines is conferred by *SACS* mutations and *PRKN* locus loss. *Conclusions:* Colorectal cancer cell lines bearing the frequent *BRAF* and *PIK3CA* mutations present many alterations of the parental cancer tissue. Described vulnerabilities represent leads for therapeutic exploration in colorectal cancers with the corresponding alterations.

## 1. Introduction

Colorectal cancer is the most prevalent gastrointestinal carcinoma and a major cause of cancer morbidity and mortality. An estimated 150,000 people will be diagnosed with colorectal cancer in 2022 in the United States alone and over 50,000 patients will die from the disease [1]. It represents the third leading cause of mortality from cancer in both men (after lung and prostate cancers) and women (behind lung and breast cancers). About 20% of cases are diagnosed in a metastatic stage and a significant percentage of initially stage II and stage III patients will have a metastatic relapse [2]. Metastatic colorectal cancer remains most often an incurable disease, despite progress in systemic and local therapies that have improved outcomes [3]. The elucidation of the molecular pathogenesis of colorectal cancer has resulted in introduction of targeted therapies that have improved survival of selected patients [4,5,6,7]. These include anti-EGFR monoclonal antibodies for *KRAS* wild type disease, combinations of anti-EGFR monoclonal antibodies with BRAF inhibitors for *BRAF* mutant cancers, anti-HER2 therapies for HER2 altered cancers and immune checkpoint inhibitors for microsatellite instability (MSI) high cancers. Other targeted treatments addressing small defined sub-sets of colorectal cancers include NTRK inhibitors for colorectal cancers with NTRK fusions and specific KRAS G12C inhibitors for cancers with this KRAS substitution [8,9]. Novel therapeutics based on combinations of targeted therapies are intensely investigated with the hope that several will enter the clinic in the near future [10,11].

*BRAF* mutations are observed in 5% to 15% of colorectal cancers and are associated with aggressive disease [12,13]. Colorectal cancers with mutations in *BRAF* tend to be of high grade and occur more often in the right colon [14]. The most common mutations in *BRAF* occur at amino-acid V600 position of the protein and substitute the normal valine at this position with glutamic acid (V600E). *BRAF* V600E mutations and other rarer substitutions at this codon location (V600K, V600D, V600M, and V600R) are categorized as class I *BRAF* mutations. These substitutions result in potent kinase activation that is independent of upstream signals from KRAS [15,16]. Mutations of *BRAF* in other codons, including the neighboring L597 and K601 positions lead to a protein that retains the requirement for homo-dimerization to signal downstream. These mutations that are classified as class II, as well as class III mutations, that require KRAS input for sustained signaling, are rare [14,15].

Mutations in the gene encoding for the alpha catalytic subunit of kinase PI3K, *PIK3CA*, are the most common colorectal cancer mutations in the PI3K/AKT/mTOR signal transduction pathway and are present in 20% to 25% of colorectal cancers [17,18,19,20]. *PIK3CA* point mutations are more diverse than *BRAF* mutations, although about half of the cases concern codons E542, E545, and Q546 of the helical domain and codon H1047 of the kinase domain. Colorectal cancers with *PIK3CA* mutations are more often arising in the right colon and present with a higher mutation count than cancers without *PIK3CA* mutations [20]. In contrast to the mutual exclusivity of mutations in oncogenes *KRAS* and *BRAF*, cancers with *PIK3CA* mutations have often concomitant mutations in either of these genes of the KRAS/BRAF/MEK/ERK pathway.

This investigation examines colorectal cancer cell lines bearing *BRAF* mutations with concomitant *PIK3CA* mutations and compares them to *BRAF* mutant cell lines without *PIK3CA* mutations in regard to genomic characteristics such as ploidy, MSI status, and coexisting molecular alterations. The sensitivity of these cell lines to drugs inhibiting the mutated pathways and to other inhibitors is also interrogated. The ultimate goal is to discover new therapeutic opportunities beyond the currently available BRAF inhibitors, which are currently the only approved drugs, in combination with anti-EGFR therapies, for colorectal cancers with V600E mutations.

## 2. Methods

Cancer cell lines included in the current investigation constitute part of the Cancer Cell Line Encyclopedia (CCLE) collection [21]. The cBioportal Genomics Portal platform was used to identify colorectal cancer cell lines with *BRAF* mutations with or without concomitant *PIK3CA* mutations in CCLE [22]. cBioportal (http://www.cbioportal.org accessed on 29 July 2022) is a user-friendly, open-access platform for genomic analysis of tumors and cancer cell lines [22]. Additionally, genomic data of colorectal cancer patients from The Cancer Genome Atlas (TCGA) study cohort [17] were analyzed using cBioportal. The CCLE project employs whole-exome sequencing to discover mutations, copy number alterations, and fusions in cell lines from various types of cancer [21]. Analysis of copy number alterations in the CCLE project was performed with the GISTIC (Genomic Identification of Significant Targets in Cancer) algorithm, in which a score of 2 or above denotes putative amplification of a gene [23]. RNA expression was normalized with the RSEM algorithm and results were presented as the Log RNA sequences in Reads per Kilobase Million (RPKM) [24].

The functional assessment of mutations observed in cell lines of interest was performed with the help of OncoKB. OncoKB knowledgebase is a database of cancer-related genes and characterizes these genes as oncogenes or tumor suppressor genes [25]. On some occasions, genes are included in OncoKB as cancer associated but they are not annotated as oncogenes or tumor suppressors.

The Genomics of Drug Sensitivity in Cancer (GDSC) dataset (www.cancerrxgene.org accessed on 29 July 2022) was interrogated to obtain data on drug sensitivity of cell lines from colorectal cancer and other cancers with *BRAF* and *PIK3CA* mutations [26]. Two datasets, GDSC1 and GDSC2, are included within the GDSC project, differing in the experimental conditions used. GDSC1 experiments were performed between 2009 and 2015. These experiments used media alone in the negative control cell lines not exposed to drugs. The GDSC2 panel of experiments was performed more recently (after 2015) and employed media with vehicle (DMSO-dimethylsulfoxide) in the negative controls. Dependencies on specific genes of cell lines with *BRAF* and *PIK3CA* mutations were obtained from the Depmap portal that contains data from CRISPR arrays and RNA-interference (RNAi) arrays of included cell lines from CCLE [27,28]. CRISPR and RNAi arrays identify essential genes that are important for the survival of screened cell lines and, as a result, the knock-down of these essential genes has a significant effect in their survival and proliferation in vitro [29,30,31]. The two methodologies differ in the depth of suppression of assayed genes, with CRISPR knock out usually being stronger than the partial suppression obtained by RNA interference. As a result, the genes and dependencies discovered with the two methodologies are not completely overlapping. Data for CRISPR screening in DepMap are from project SCORE containing 323 cancer cell lines from various cancers and a library of 18,009 targeted genes [32]. Computational modelling of experiments in SCORE was initially performed with the CERES algorithm and later with the CHRONOS algorithm [33,34]. RNAi experiments were performed under the aegis of project Achilles using the DEMETER algorithm for analysis [30].

Statistical comparisons of categorical data were carried out using Fisher’s exact test or the x^2^ test. The Mann–Whitney U test was used to compare median values. All statistical comparisons were considered significant if *p* < 0.05.

All data presented in this paper are from experiments performed by the consortiums mentioned in the above methods section and are openly available in the public domain. No new laboratory experiments have been performed for this investigation.

## 3. Results

The colorectal cancer cohort of CCLE consisting of 84 cell lines contains 23 cell lines (27.4%) with *BRAF* mutations. Ten *BRAF* mutant cell lines contain classic V600E mutations, in three of them (OUMS23, MDST8 and HT-29) with additional non-canonical *BRAF* mutations (Table 1). Thirteen cell lines contain non-V600E mutations. In two of them, NCI-H508 and HT-55, mutations are oncogenic or potentially oncogenic (G596R and N581Y, respectively).

Seven *BRAF* V600E mutant cell lines are wild type for *PIK3CA*, while three cell lines with V600E mutations (SNU-C5, RKO and HT-29) as well as cell line NCI-H508, which has a pathogenic non-V600 mutation at position G596, have concomitant pathogenic mutations in *PIK3CA* (Table 1). Five of the seven cell lines with V600E *BRAF* mutations and no *PIK3CA* mutations are MSS, possess a lower mutation count, are hyper-diploid and have a high Fraction of Genome Altered (FGA) (Table 2). The two V600E *BRAF* mutant/*PIK3CA* wild type colorectal cancer cell lines, LS411N and CL34, that are MSI high have consistently a high mutation count. The two cell lines with concomitant *BRAF* V600E and *PIK3CA* H1047R mutations, SNU-C5 and RKO, are MSI high, have a high mutation count, are diploid and have a low FGA (Table 2). The two other cell lines with concomitant mutations, NCI-H508 and HT-29, have non-canonical pathogenic mutations in either *BRAF* (NCI-H508) or in *PIK3CA* (HT-29) and they are both MSS, have lower mutation counts, are hyper-diploid and have a high FGA.

Regarding concomitant cancer-associated mutations in V600E *BRAF* mutant/*PIK3CA* wild type colorectal cancer cell lines all seven cell lines have oncogenic mutations in *APC* and four have also oncogenic mutations in *TP53* (Table 3). No cell lines have *KRAS* mutations, which tend to be mutually exclusive with *BRAF* mutations. Recurrent oncogenic deletions include the loci of dual specificity phosphatase *DUSP22*, which is present in 4 cell lines and deletions in *SMAD4* and *SMAD2*, which are present in 3 and 2 cell lines, respectively (Table 3). Only two of the four cell lines with oncogenic mutations in both *BRAF* and *PIK3CA* have concomitant *APC* mutations and three of the four have also *TP53* mutations (Table 3). Recurrent amplifications are observed in *MYC* and *AGO2* that are both located at chromosome arm 8q and are present in cell lines RKO and HT-29. These cell lines and the cell line NCI-H508 also possess deletions of *PRKN*, encoding for ubiquitin ligase parkin, which is the only recurrent deletions in *BRAF*/*PIK3CA* double mutant colorectal cancer cell lines. HT-29 is the only double mutant cell line possessing the recurrent deletion of *DUSP22*, observed in cell lines with V600E *BRAF* mutations and wild type *PIK3CA* (Table 3).

Vulnerabilities of *BRAF* mutant cell lines with or without *PIK3CA* mutations were explored with interrogation of RNAi libraries for determination of preferentially essential genes and with CRISPR mediated knock out arrays (Table 4). Recurrent genes that are observed to be essential for survival in more than one *BRAF* mutant cell lines include *CTNNB1*, encoding for β-catenin, *WRN*, encoding for Warner syndrome ATP-dependent helicase, *ALYREF* which encodes for a chaperone of basal region leucine zipper (bZIP) proteins, and peptidylprolyl isomerase E (PPIE). These recurrent essential genes are in the top list of preferentially essential genes in one or more of the four cell lines with *BRAF* and *PIK3CA* mutations (Table 4). In addition, the gene encoding for CAD, an enzyme of the pyrimidine biosynthesis pathway induced by MAPK cascade, is a preferentially essential gene in two of four *BRAF* and *PIK3CA* mutant cell lines.

**Figure 1:**
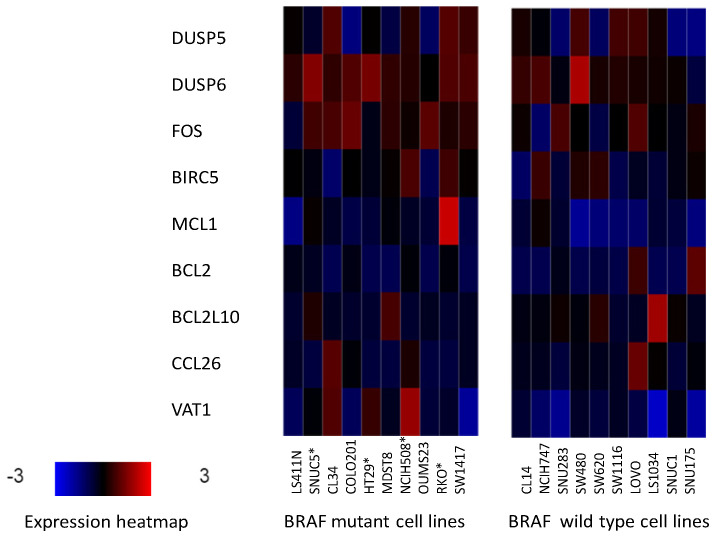
mRNA expression of genes targeted by the BRAF/MEK/ERK pathway (DUSP5, DUSP6, FOS, BIRC5, and MCL1) and genes not directly targeted by the BRAF/MEK/ERK pathway (BCL2, BCL2L10, CCL26 and VAT1) as controls in representative colorectal cancer cell lines with (left panel) and without (right panel) mutations in *BRAF*. BRAF mutated cell lines with coexisting PIK3CA mutations are shown with an asterisk.

Five of the seven cell lines with *BRAF* mutations and without *PIK3CA* mutations (COLO205, MDST8, LS411N, SW1417 and CL34) have been assayed for drug sensitivities in GDSC (Table 5). Top drug sensitivities displayed by cell lines COLO205 and CL34 are to BRAF inhibitors, inhibitors of downstream MEK kinases and inhibitors of upstream receptor tyrosine kinases. LS411N cell line displays sensitivity to drugs of the pathway as well as to other kinases and the dihydrofolate reductase inhibitor pyrimethamine. In contrast, no inhibitors of BRAF or the receptor tyrosine kinase/KRAS/BRAF/MAPK pathway are among the top sensitivities of cell lines MDST8 and SW1417. Top sensitivities of these two cell lines include drugs involved in lipid metabolism and apoptosis inhibitors (Table 5). Cell lines with mutations in both *BRAF* and *PIK3CA* display sensitivities to several inhibitors of the receptor tyrosine kinase/KRAS/BRAF/MAPK pathway and PI3K/AKT cascade. Two of the four *BRAF*/*PIK3CA* double mutated cell lines, SNUC5 and RKO present additional sensitivities to the clinically used antimetabolite methotrexate, the WEE1 kinase inhibitor MK-1775, the mitotic kinases AURKA and AURKB inhibitor ZM447439 and the epigenetic modifier, BET bromodomain inhibitor JQ1. Compared with cell lines not bearing mutations in *BRAF* and *PIK3CA*, colorectal cancer cell lines with *BRAF* mutations with or without *PIK3CA* mutations show heterogeneous up-regulation in the mRNA expression of genes that are targets of the BRAF/MEK/ERK pathway. These include phosphatases DUSP5, DUSP6, AP-1 transcription factor component FOS, and apoptosis inhibitors survivin (also known as BIRC5—that is, baculoviral IAP repeat containing 5) and MCL1 (Figure 1). However, the robustness of pathway upregulation as suggested by the upregulation of these genes does not correlate with sensitivity to BRAF inhibitors. For example, cell lines SW1417 and MDST8, which display upregulation of pathway target genes, show no BRAF or other pathway inhibitors among their top inhibiting drugs (Table 5).

GDSC includes five specific BRAF inhibitors among the panel of assayed drugs. Recurrent molecular characteristics of the colorectal cancer cell lines panel that confer sensitivity to specific BRAF inhibitors include, as expected, *BRAF* mutations conferring sensitivity to 4 of the 5 inhibitors (Table 6). In addition, the presence of *KRAS* mutations confer resistance to 3 of the 5 BRAF inhibitors, as they tend to be mutually exclusive with *BRAF* mutations and segregate with *BRAF* wild type cell lines. Another genomic feature that is present recurrently among the abnormalities conferring BRAF inhibitor sensitivity in colorectal cancer cell lines is mutations in SACS, a gene encoding for sacsin, a chaperone protein. The most common copy number alteration that confers resistance to 3 of the 5 BRAF inhibitors is a loss at chromosome 6q26, a locus containing gene *PRKN*, encoding for E3 ubiquitin ligase parkin (feature cnaCOREAD24). Loss of *PRKN* is a feature of some *BRAF* mutant cell lines, as mentioned above, and it is also, rarely, encountered in *BRAF* mutant colorectal cancers. Thus, resistance to BRAF inhibitors associated with concomitant loss of *PRKN* may be of clinical significance. Interestingly, *PIK3CA* mutations do not feature among the molecular abnormalities conferring resistance to specific BRAF inhibitors in colorectal cancer cell lines. The only BRAF specific inhibitor that is not significantly more effective in *BRAF* mutant cell lines is HG6-64-1, which displays a separate private panel of mutations conferring resistance, not observed in other BRAF inhibitors. These include EGFR mutations and mutations in kinase ATM (Table 6).

In the pan-cancer analysis of cell lines with *BRAF* mutations, which is more statistically robust due to the number of cell lines assayed, pathway inhibitors (BRAF inhibitors: Dabrafenib, PLX-4720, SB59088, MEK inhibitors: selumetinib, trametinib, refametinib, PD0325901, ERK inhibitors: ulixertinib, ERK2440, ERK6604, SCH772984, VX-11e) are significantly associated with sensitivity compared to cell lines without *BRAF* mutations. In addition, the inhibitor of NUAK1 and NUAK2 kinases WZ4003 is statistically significantly associated with sensitivity in BRAF mutant cell lines compared with *BRAF* wild type cell lines (IC50 effect size: −0.34, *p* = 8.03 × 10^−5^). Specifically for colorectal cancer cell lines, *BRAF* mutant cell lines display also greater sensitivity to inhibitor WZ4003 compared to *BRAF* wild type colorectal cancer cell lines (mean IC50: 63.7 μM versus 132 μM), although, due to smaller numbers, this difference did not reach statistical significance (*p* = 0.08).

## 4. Discussion

BRAF is an oncogenic serine/threonine kinase, which is mutated in various cancers, most commonly in melanoma, thyroid carcinomas, hairy cell leukemia, lung cancers, and colorectal cancers [35]. The gene encoding for the kinase is located on the human chromosome locus 7q34. BRAF is activated by KRAS downstream of growth factor receptors and activates the Mitogen Activated Protein Kinase (MAPK)/Extracellular signal-Regulated Kinase (ERK) signaling cascade promoting cell proliferation. The importance of this pathway in cancer is highlighted by the fact that KRAS is the most frequently mutated oncogene across cancer types [36]. In parallel with the KRAS/BRAF/MAPK/ERK pathway, and also activated by growth factor receptors, the PI3K/AKT/mTOR cascade plays an important role in carcinogenesis through inhibition of apoptosis, cell growth promotion and oncogene activation [37]. *PIK3CA*, the gene encoding for the catalytic alpha sub-unit of kinase PI3K is often mutated in prevalent cancers such as breast cancer and colorectal adenocarcinomas. In colorectal cancer, *PIK3CA* is mutated in 20% to 25% of cases and is the second most commonly mutated oncogene after *KRAS* [17]. *BRAF* mutated colorectal cancers are less prevalent, representing 5% to 15% of all colorectal cancers. Most of *BRAF* mutations are located at amino acid position V600, substituting glutamic acid for valine that is normally at this position in the wild type protein (V600E substitution). Substitutions at position V600 render the protein independent from KRAS and result in robust kinase-mediated activation of MAPK cascade, without the physiologic input from growth factors [38]. Other less common *BRAF* mutations produce a protein with lower kinase activity or even a kinase-dead protein that can still activate down-stream signaling through interaction with the homologous CRAF kinase [15]. Canonical V600E BRAF mutations are mutually exclusive with *KRAS* mutations. In contrast, *PIK3CA* mutations are encountered in colorectal cancers with either *KRAS* or *BRAF* mutations with an equal or higher prevalence than in cancers with wild type *KRAS* and *BRAF*.

*BRAF* mutations are targeted currently in colorectal cancer in the clinic at the second line metastatic setting with a regimen that combines BRAF inhibitors and anti-EGFR monoclonal antibodies. This combination has provided superior efficacy and survival outcomes compared with chemotherapy, with a modest improvement of 3 months in Overall Survival (OS) [39]. In contrast, no therapies targeting *PIK3CA* mutated colorectal cancers have been approved for clinical use. Combinations of BRAF inhibitors with PI3K inhibitors have not been studied in a systematic manner in colorectal cancer, but few available retrospective data suggest that parallel inhibition of the two mutated oncogenes may provide a synergistic effect in double mutant cancers [40]. Unveiling vulnerabilities of colorectal cancers with *BRAF* mutations with and without concomitant *PIK3CA* mutations may provide new opportunities for targeted treatments.

The current investigation examines a panel of colorectal cancer cell lines with *BRAF* mutations with or without concomitant mutations in *PIK3CA* from the CCLE for drug sensitivities and molecular dependencies. Mutations in *PIK3CA* are the most frequent mutations in the receptor tyrosine kinase-initiated pathways in colorectal cancers with *BRAF* mutations, as the even more frequent *KRAS* mutations are mutually exclusive with *BRAF* mutations. Colorectal cancer cell line models recapitulate the presence of *BRAF* and *PIK3CA* mutations as encountered in clinical colorectal cancer samples, and also duplicate the frequent presence of MSI in these cases [41]. Mutations in tumor suppressors *APC* and *TP53* are often present in *BRAF* mutant colorectal cancer cell lines, similar to clinical samples. Cell lines with *BRAF* mutations and wild type *PIK3CA* possess also deletions of signal transducers of TGFβ pathway SMAD4 and SMAD2 and of phosphatase DUSP22. The genes of these proteins are rarely deleted in clinical colorectal cancer, but they are more commonly mutated. For example, in TCGA cohort, *SMAD4* mutations are observed in 16.1% of cases with *BRAF* mutations, *SMAD2* mutations are observed in 6.5% of cases with *BRAF* mutations and *DUSP22* mutations are encountered in 9.7% of patients with *BRAF* mutations [17]. The presence of mutations or deletions of these genes suggest that decreased availability and function of the resulting proteins may be essential for *BRAF* mutant cancers both in vitro and in vivo. The TGFβ signaling pathway and tumor suppressor *SMAD4* mutations have been implicated in the serrated colon carcinogenesis pathway commonly resulting from *BRAF* mutations [42]. In addition, inhibitors of the TGFβ receptor TGFBR1 prevented the development of resistance to BRAF inhibitor vemurafenib in *BRAF* mutant melanoma cells [43]. Thus, inhibitors of the TGFβ pathway, should they become clinically available, could be candidates for combination therapies in *BRAF* mutated colorectal cancers. Phosphatase DUSP22 (also called JKAP- c-JUN N-terminal Kinase Associated phosphatase) is a regulator of the MAPK pathway, and as a result, it may modulate the effect of *BRAF* mutations in the pathway output [44]. DUSP22 showed lower mRNA expression in colorectal cancer tissues compared to adjacent normal colonic mucosa [45]. In this study that included 92 patients, patients with metastatic colorectal cancer and low expression of DUSP22 had a trend towards worse survival, although not statistically significant [45].

The analysis of molecular features associated with sensitivity or resistance to BRAF specific inhibitors reveals that, besides *BRAF* mutations and *KRAS* mutations that are associated with sensitivity and resistance to the drugs, respectively, no other abnormalities of the pathway affect sensitivity to these drugs in a consistent manner, in vitro. Unrelated molecular alterations associated with sensitization of colorectal cancer cell lines to BRAF inhibitors included mutations in *SACS*, encoding for chaperone protein sacsin and deletions at the locus of parkin. Sacsin is a large protein with chaperone function in the nervous system and loss of function mutations are associated with the degenerative disorder autosomal recessive spastic ataxia of Charlevoix-Saguenay [46]. Cells with sacsin loss of function have defective mitochondrial dynamics and increased oxidative stress. Mutations in *SACS* have not been previously linked with colorectal cancer. The protein consists of 4579 amino acids and is mutated in 12.5% of colorectal cancers of the TCGA cohort with mutations distributed equally across the length of the protein [17]. It is also mutated in 33.9% of colorectal cancers with *BRAF* mutations and in 19% of cancers with *PIK3CA* mutations. Among colorectal cancers classified as MSI high or with proofreading polymerase epsilon mutations, *SACS* mutations are present in 42.5% of cases, suggesting that these mutations are associated with high TMB and may be passenger [47]. Alternatively, an oncogenic role of sacsin mutations in colorectal cancer is also possible based on its function in oxidative stress and deserves to be formally confirmed or excluded.

Concomitant mutations in *APC* that are observed in most cell lines with *BRAF* mutations with or without *PIK3CA* mutations, as well as the fact that *CTNNB1* gene, encoding for β-catenin, is a recurrent preferential essential gene in these cell lines suggest that *BRAF* mutated colorectal cancers remain dependent on the activity of WNT/APC/β-catenin pathway [48,49]. Two other recurrent preferentially essential genes in *BRAF* mutated cell lines are *WRN*, encoding for Werner helicase and *CAD* (carbamoyl-phosphate synthetase 2, aspartate transcarbamylase and dihydroorotase), encoding for a protein with trifunctional enzyme activity implicated in the de novo pyrimidine nucleotide biosynthesis. WRN helicase is involved in DNA repair and was recently identified as a vulnerability of cancer cells with MSI [27,50,51,52]. Cells with MSI are vulnerable to massive apoptosis in the absence of WRN function because of accumulation of long TA dinucleotide repeats that form secondary structures that stall DNA forks during replication [53]. Consistent with this mechanism, MSS cell lines are not dependent on WRN helicase function [52]. Indeed, the *BRAF* mutant colorectal cancer cell lines that show vulnerability to WRN knock-down are all MSI high, suggesting that this is the underlying molecular defect directly responsible, rather than *BRAF* mutations. However, given the frequent co-occurrence of the two alterations in cell lines and clinical colorectal cancers, pharmacologic inhibition of WRN helicase in these cancers can be envisioned and would be expected to spare normal cells without MSI.

The other recurrent preferentially essential gene discovered in *BRAF* mutated cell lines, CAD, possesses the three first enzymatic activities in the pathway of de novo pyrimidine nucleotide biosynthesis in a single polypeptide of 2225 amino acids [54]. CAD is regulated by phosphorylation by MAPK, which activates the enzyme to promote nucleotide synthesis [55]. This regulation makes CAD a target of the KRAS/BRAF/MAPK cascade in response to growth factor signaling and activates an enzymatic function that sustains nucleotide production required for cell proliferation. Moreover, in colorectal cancer, CAD is regulated by MYC and when the metabolic reprogramming observed in cancer cells as a result of MYC activation is inhibited, cell growth is blocked by shutting down CAD and other enzymes of pyrimidine biosynthesis [56]. In cancer cells with deregulated proliferation secondary to *BRAF* mutations, loss of CAD function would deprive them from the required de novo pyrimidine nucleotides with potential catastrophic consequences due to loss of the coordinated response to the metabolic needs derived by high cancer cell proliferation. Thus, pharmacologic CAD inhibition with novel inhibitors in development may represent a therapeutic target in *BRAF* mutated cells with concomitant *PIK3CA* mutations, given that MAPK signaling and MYC are regulated by the two oncogenes [57].

A final interesting finding of the current investigation with potential future therapeutic implications is the identification of a NUAK family kinase (NUAK) inhibitor as one of the top hits in the pan-cancer BRAF mutant cell line screening. NUAK1 and NUAK2 are AMPK (AMP-activated Protein Kinase) related kinases with diverse functions in cancer cells [58]. NUAK1 promotes motility, invasion, and metastases of cancer cells [59,60]. NUAK1 shows higher expression in advanced stage colorectal cancers and in biopsies from liver metastatic sites, compared to primary tumors [61]. An important role of the kinase has been described in cancer cells with oncogene MYC overexpression, related to protection from oxidative stress resulting from MYC activity [62]. Mechanistically, NUAK1 contributes to mitochondrial plasticity and adaptation which is critical for cells bearing induction of oxidative respiratory chain component proteins effectuated by MYC [63]. Only 2 colorectal cancer cell lines with *BRAF* mutations RKO and HT-29 show MYC amplifications and both are more sensitive to the NUAK inhibitor WZ4003 than the mean sensitivity of the *BRAF* mutant group of colorectal cancer cell lines. Although these observations are based on a small number of cell lines, they suggest that *BRAF* mutant colorectal cancers with concomitant aberrations increasing oxidative stress could be candidates for combination therapies with NUAK kinases inhibitors.

A limitation of the current study is that relies exclusively in in silico publicly available data and no further experimental confirmation was performed. In addition, in the drug sensitivity analysis based on GDSC, cell lines are exposed to the assayed drugs as monotherapies and no data exist to inform combination therapies. Combinations of targeted anti-neoplastic drug therapies are increasingly recognized as being necessary for improvement of response in cancers which accumulate molecular alterations over time for their survival. Another limitation of the current study is that the cell line data do not definitely allow differentiation of a direct dependency on *BRAF* or *PIK3CA* mutations versus indirect effects related to other vulnerabilities such as MSI commonly co-occurring in these cell lines as the example of WRN helicase dependency illustrates. Moreover, it is expected that additional vulnerabilities that are not revealed with the approach used here exist in *BRAF* mutant colorectal cancers. For example, RANBP2, a binding protein of RAN (RAS related nuclear protein), a small GTPase of the RAS family, has been proposed as essential for survival of *BRAF* V600E mutant colorectal cancer cells and cells with a similar genomic signature [64].

In conclusion, targeted therapies of colorectal cancers that possess *BRAF* mutations with or without *PIK3CA* mutations could be developed based on the global molecular environment of these cancers and based on vulnerabilities uncovered in in vitro models. It is reassuring for the validity of the vulnerabilities discovered from cell lines models, that some of them, such as, for example, the synthetic lethality of MSI and WRN helicase, had previously been reported in pertinent systems. Leads discussed here need to be confirmed in in vivo studies followed by human trials in the population of interest.

## Figures and Tables

**Table 1:** *BRAF* mutated colorectal cancer cell lines and their specific *BRAF* mutations and concomitant *PIK3CA* mutations. Data are from the Cancer Cell Line Encyclopedia (CCLE). WT: wild type.

Cell Line	*BRAF*	*PIK3CA*
*BRAF* V600E mutations		
COLO205	V600E	WT
COLO201	V600E	WT
LS411N	V600E	WT
SW1417	V600E	WT
CL34	V600E	WT
MDST8	V600E, V600K, V600M	WT
OUMS23	V600E, X287_splice	WT
SNU-C5	V600E	H1047R
RKO	V600E	H1047R
HT-29	V600E, T119S	P449T
*BRAF* non-V600E pathogenic mutations		
HT-55	N581Y	WT
NCI-H508	G596R	E545K
*BRAF* mutations of unknown significance		
HT115	R354Q	R88Q, E321D, R770Q
SNU-C4	D22N	E545G, V71I
CCK81	S273N, R506G	C420R, C472Y
LS513	E204L, E204V, E204*	WT
GP2D	T529A	H1047L
SNU1040	V120I, S76P	L632*
SNU407	R726C	H1047R
SNU503	D22N	WT
LS180	D211G	H1047R
KM12	A712T, A404Cfs*9	WT
GP5D	T529A	H1047L

**Table 2:** Characteristics of colorectal cancer cell lines with *BRAF* V600E mutations without and with concomitant *PIK3CA* mutations. Cell line NCI-H508 has a *BRAF* G596R pathogenic mutation instead of *BRAF* V600E mutation. Cell lines without an asterisk are without *PIK3CA* mutations and are presented first. Cell lines with an asterisk in the bottom lines of the table are those with concomitant *PIK3CA* mutations.

Cell Line	DepMap ID	Mutation Count	FGA	Ploidy	MSI Status
COLO205	ACH-001039	307	0.44	3.2	MSS
OUMS23	ACH-000296	340	0.50	2.5	MSS
COLO201	ACH-000253	255	0.38	2.96	MSS
MDST8	ACH-000935	776	0.55	3.82	MSS
LS411N	ACH-000985	5442	0.28	3.30	MSI
SW1417	ACH-000236	248	0.56	3.01	MSS
CL34	ACH-000895	1280	0.14	1.95	MSI
SNUC5 *	ACH-000970	2990	0.09	2.0	MSI
RKO *	ACH-000943	3424	0.14	2.1	MSI
NCI-H508 *	ACH-000360	318	0.48	4.6	MSS
HT-29 *	ACH-000552	416	0.43	3.04	MSS

**Table 3:** Molecular alterations in colorectal cancer cell lines with *BRAF* V600E mutations without and with concomitant *PIK3CA* mutations. +: presence of oncogenic mutation. Cell lines with an asterisk are those with concomitant *PIK3CA* mutations.

Cell Line	APC	TP53	KRAS	SMAD4	ATM	FBXW7	Other Mutations	Amplifications	Deletions
COLO205	+							CCND3	CDC73, DUSP22, SMAD4
OUMS23	+	+					TBX3	AURKA, YES1	PTEN, MAP2K4, CDC73, FAT1, SMAD4, SMAD2, BMPR1A
COLO201	+						EPHA7, BACH2		
MDST8	+						CDKN2A		DUSP22, HLA-A, PAX5, BCL11B
LS411N	+	+				+	BARD1, BRIP1, PTEN, ARID1A, RNF43, MLH1, KMT2A, KMT2A, KMT2D	FGFR1	HLA-A, SMAD4, SMAD2, PMAIP1
SW1417	+	+					RTEL	MET, AURKA, BRAF, SRC, BCL2L1, EZH2, RHEB, DNMT3B	IKZF1, FLCN, NKX3-1, DUSP22, PIK3R1, PPP2R2A
CL34	+	+					TSC1, HLA-B, TCF7L2, PPM1D, PARP1, TP53BP1, CREBBP, TGFBR2, AMER1, ATRX, AXIN2, SOX9, ARID4B, MAP2K4		DUSP22, FOXP1, H1-3, JARID2,
SNUC5 *	+	+			+	+	ERCC2, ARID1A, CTCF, ARID2, RNF43, PPM1D, DNMT3A, ZFHX3, CREBBP, CYLD, EP300, LATS1, KMT2C, FANCC, KMT2B, ARID4A, NCOR1, ASXL2, KMT2D, BCORL1, CD58, ELF3, MED12, EP400	AR	FLCN, PTPRT
RKO *							BRCA2, ARID1A, NF1, STAT3, KMT2A, B2M, BCORL1, EP300, RNF43, JARID2, NCOR1, PARP1, TET1, TP53BP1, CREBBP, FANCA, NOTCH3, NF2, NSD1, PTPRD, MSH6, FAT1, GATA3, SOX9	UBR5, AGO2, MYC	PTPRD, PRKN, PAX5, FOXA1, EP300, INHA
NCI-H508 *		+					CDKN2A, SPOP, CREBBP	PIK3CA, BCL2L1, BCL6, DNMT3B, CDK8	PRKN, INPP4B, TCF7L2
HT-29 *	+	+		+			CASP8, SLFN11	KIT, AGO2, MYC, CDK8	NKX3-1, DUSP22, PRKN, ESCO2, PPP2R2A, EPHA3

**Table 4:** Top dependencies of *BRAF* V600E mutant/*PIK3CA* wild type and *BRAF* V600E mutant/*PIK3CA* mutant colorectal cancer cell lines, as determined by RNAi and CRISPR knock-out. RNAi experiments are from project Achilles and CRISPR experiments are from project SCORE and CHRONOS. NA: not available.

Cell Line	Top 10 Preferentially Essential Genes RNAi	Top CRISPR KO Genes
COLO205	NA	YRDC, ADSL, MMS22L, UMPS, TRNT1 (SCORE)
OUMS23	GSPT1, ALYREF, BUB3, BUB1B, RPL13, PHB, QARS1, SERPINA5, MAD2L1,ZRSR2	SLC25A37, SCAP, ATP6V1A, RGP1, SOD2, CHAF1B, CIAO2B, CHAF1A, ATP6VOB, DHX9 (CHRONOS)
COLO201	BRAF, MAP2K1, MYBL2, BCL2L1, CTNNB1, MAPK1, SOX9, CHD4, TCF7L2, PSMD2	PSMG4, MYB, CLNS1A. HSPA8, RUVBL1 (SCORE)CTNNB1, DUSP4, HSPA8, WASF2, SOX9, SLC1A5, NIBAN2, BRAF, ASCL2, IQGAP1 (CHRONOS)
MDST8	NA	HSPD1, USP17L5, MDM2, YRDC, CYCS (SCORE)
LS411N	CTNNB1, WRN, DDX39A, BCL2L1, ALYREF, DHX9, JPT2, PCNA, PPIE	TINF2, RNPC3, NXT1, HSPA9, NUP85 (SCORE)NXT1, SLC7A1, CTNNB1, DDX39A, WRN, BCL2L1, INTS6, ADSL, SYNCRIP, SNAP23 (CHRONOS)
SW1417	CDC40, PPIE, KHSRP, EFCAB8, PPP2CA, PPWD1, EIF4A3, MED11, OR56B1, CAPZB	NA
CL34	CTNNB1, WRN, ZNF432, SCAP, CWC22, NUP214, TTC1, RPA3, SAP130, PHB	NA
SNUC5	NA	CDCA8, YRDC, WDR82, FAU, BUD31 (SCORE)WRN, NAMPT, TRPM7, RFK, ADSL, PELO, NDE1, MTHFD1, PPIE, RAB1 (CHRONOS)
RKO	OGDH, WRN, ALDH18A1, URI1, RPL22L1, CAD, TTC7A, CD3EAP, SDHD, SDHC	CCT4, DYNLRB1, UBE2M, FAU, RPP21 (SCORE)ATPV0E1, WRN, CREBBP, TTC7A, CAD, SLC5A3, MTCH2, MTX2, UMPS, FAM126B (CHRONOS)
NCI-H508	MYBL2, SLC22A20P, TYMS, ANKRD2019P, SKP1, PSMA3, CTNNB1, YAP1, EFCAB8, EGFR	NA
HT-29	RAB6A, GINS2, APC, AHCTF1, BRAF, COP1, CAD, PFAS, SUMO2	DYNLRB1, THAP1, MYC, PPP2CA, INTS6 (SCORE)PTDSS1, INTS6, RIC1, SCD, SCAP, MBTPS2, NDE1, STX4, RAB10, HNF1B (CHRONOS)

**Table 5:** Drug sensitivities of *PIK3CA* wild type/*BRAF* V600E mutant cell lines. Data are from the Genomics of Drug Sensitivity in Cancer (GDSC).

Cell Line	Drug	Target	IC50	Z Score	Source
COLO205	SB590885	BRAF	0.18	−4.13	GDSC1
	PLX-4720	BRAF	0.19	−4.04	GDSC1
	Selumetinib	MEK1, MEK2	0.08	−3.39	GDSC1
	BMS-754807	IGF1R, IR	0.01	−3.17	GDSC1
	Lisitinib	IGF1R	0.09	−3.15	GDSC2
MDST8	CAY10566	Steroyl-CoA Desaturase	0.07	−4.20	GDSC1
	SGC0946	DOT1L	0.99	−3.03	GDSC1
	CCT007093	PPM1D	7.7	−2.76	GDSC1
	UNC1215	L3MBTL3	2.37	−2.61	GDSC1
	(5Z)-7-Oxozeanol	TAK1	0.04	−2.57	GDSC1
LS411N	Pyrimethamine	Dihydrofolate reductase	0.72	−2.82	GDSC1
	VX11e	ERK2	0.56	−2.33	GDSC1
	AZ628	BRAF	0.11	−2.13	GDSC1
	Alectinib	ALK	3.98	−2.12	GDSC1
	GNF-2	BCR-ABL	2.20	−2.01	GDSC1
SW1417	WEHI-539	BCL-XL	0.33	−2.48	GDSC2
	Sphingosine kinase 1 inhibitor II	Sphingosine kinase	10.2	−2.05	GDSC1
	CHIR-99021	GSK3A, GSK3B	3.07	−1.99	GDSC1
	Navitoclax	BCL2, BCL-XL, BCL-W	0.28	−1.61	GDSC2
	SN-38	TOP1	0.00	−1.43	GDSC1
CL34	Trametinib	MEK1, MEK2	0.00	−2.92	GDSC2
	Dabrafenib	BRAF	0.16	−2.71	GDSC2
	SCH772984	ERK1, ERK2	0.06	−2.61	GDSC2
	Selumetinib	MEK1, MEK2	0.06	−2.34	GDSC1
	PLX-4720	BRAF	2.77	−2.01	GDSC1
SNUC5	Methotrexate	Antimetabolite	0.04	−1.52	GDSC1
	PD0325901	MEK1, MEK2	0.04	−1.20	GDSC1
	Bosutinib	SRC, ABL	1.16	−1.18	GDSC1
	PLX-4720	BRAF	13.55	−1.13	GDSC1
	MK-1775	WEE1	0.48	−1.12	GDSC1
RKO	KIN-001	GSK3B	13.4	−2.7	GDSC1
	Selumetinib	MEK1/2	0.29	−2.49	GDSC1
	AZ628	BRAF	0.06	−2.47	GDSC1
	ZM447439	AURKA/B	0.58	−2.19	GDSC1
	JQ1	BRD2/3/4	0.05	−2.13	GDSC1
NCI-H508	Afatinib	ERBB2, EGFR	0.04	−2.81	GDSC1
	Afatinib	ERBB2, EGFR	0.07	−2.71	GDSC2
	Gefitinib	EGFR	0.23	−2.12	GDSC1
	Pictilisib	PI3K (class 1)	0.18	−2.00	GDSC1
	MK-2206	AKT1, AKT2	0.87	−1.97	GDSC2
HT-29	ERK_6604	ERK1, ERK2	0.62	−2.20	GDSC2
	BMS-754807	IGF1R, IR	0.05	−2.17	GDSC1
	Linsitinib	IGF1R	0.42	−2.08	GDSC1
	Refametinib	MEK1, MEK2	0.13	−1.98	GDSC1
	AS605240	PI3Kgamma	1.04	−1.98	GDSC1

**Table 6:** Top molecular features with increased sensitivities to various BRAF inhibitors (statistically significant or approaching significance). Two non-specific RAF inhibitors (RAF 9304 and Sorafenib) are also shown. Data are from the Genomics of Drug Sensitivity in Cancer (GDSC).

Drug	Feature	IC50 Effect Size	*p* Value	Number of Altered Cell Lines	Dataset
AZ628	SACS mutation	−2.45	0.007	3	GDSC1
	cnaCOREAD19	−2.05	0.008	4	GDSC1
	BRAF mutation	−1.52	0.04	5	GDSC1
	KRAS mutation	−0.44	0.08	6	GDSC1
	FBXW7 mutation	−1.26	0.08	3	GDSC1
Dabrafenib	BRAF mutation	−2.24	2.21 × 10^−7^	10	GDSC2
	KRAS mutation	0.85	0.006	24	GDSC2
	cnaCOREAD24	0.99	0.012	9	GDSC2
	KDM6A mutation	1.21	0.016	3	GDSC2
	cnaCOREAD55	0.89	0.02	10	GDSC2
	cnaCOREAD56	0.89	0.02	10	GDSC2
	SACS mutation	−0.82	0.031	10	GDSC2
HG6-64-1	ATM mutation	1.33	0.02	3	GDSC1
	SMARCA4 mutation	0.41	0.033	3	GDSC1
	EGFR mutation	0.39	0.037	3	GDSC1
	PBRM1 mutation	1.18	0.039	3	GDSC1
PLX-4720	BRAF mutation	−1.77	1.58 × 10^−5^	10	GDSC2
	KRAS mutation	0.91	0.002	25	GDSC2
	SACS mutation	−1.03	0.009	10	GDSC2
	cnaCOREAD19	−0.83	0.013	18	GDSC2
	cnaCOREAD55	0.88	0.018	11	GDSC2
	cnaCOREAD56	0.88	0.018	11	GDSC2
	cnaCOREAD23	1.46	0.026	3	GDSC2
	cnaCOREAD53	1.46	0.026	3	GDSC2
	cnaCOREAD24	0.84	0.034	9	GDSC2
	BCOR mutation	−1.05	0.045	5	GDSC2
SB590885	BRAF mutation	−1.21	0.002	10	GDSC1
	cnaCOREAD24	1.16	0.004	9	GDSC1
	KRAS mutation	0.8	0.009	25	GDSC1
	cnaCOREAD12	1.65	0.012	3	GDSC1
	SACS mutation	−0.95	0.018	10	GDSC1
	cnaCOREAD56	0.83	0.029	11	GDSC1
	cnaCOREAD55	0.83	0.029	11	GDSC1
RAF 9304	cnaCOREAD63	1.25	0.009	6	GDSC1
(pan-RAF)	TP53 mutation	0.96	0.009	33	GDSC1
	ARID1B mutation	1.17	0.036	3	GDSC1
	PIK3R1 mutation	1	0.047	5	GDSC1
	BRAF mutation	−0.76	0.047	10	GDSC1
	NCOR1 mutation	−0.95	0.049	6	GDSC1
Sorafenib	cnaCOREAD47	−0.89	0.01	6	GDSC2
(PDGFR, c-KIT,	KDM6A mutation	1.05	0.01	6	GDSC2
VEGFR, RAF)	cnaCOREAD48	1.24	0.026	5	GDSC2
	CEP290 mutation	−1.09	0.029	5	GDSC2
	KRAS mutation	0.63	0.037	24	GDSC2
	cnaCOREAD14	1.19	0.049	3	GDSC2

## Data Availability

There are no data available beyond data included in the manuscript.
